# Inducing factors and complication risks of pelvic inflammatory disease: a review

**DOI:** 10.3389/fendo.2026.1843375

**Published:** 2026-06-22

**Authors:** Guanglong Wang, Suo Zhang

**Affiliations:** College of Traditional Chinese Medicine, Inner Mongolia Medical University, Hohhot, China

**Keywords:** complications, inducing factors, pelvic inflammatory disease (PID), reproduction, reproductive health

## Abstract

Pelvic inflammatory disease (PID) is a common infectious condition of the female upper genital tract that imposes a substantial global burden on women’s reproductive health and overall quality of life. This narrative review aims to comprehensively synthesize current evidence on the inducing factors and complication risks of PID. We conducted a systematic literature search in PubMed, Embase, and Web of Science databases from January 2010 to December 2025, prioritizing high-quality evidence including systematic reviews, meta-analyses, large-scale population-based cohort studies, and national registry data. The review systematically categorizes PID etiologies into sexually transmitted infections, cervicovaginal dysbiosis, iatrogenic procedures (intrauterine device insertion, assisted reproductive technology, and intrauterine manipulations), anatomical abnormalities, underlying gynecological conditions, lifestyle habits, and environmental exposures, with a particular focus on the emerging role of cervicovaginal microbiota in idiopathic PID. Beyond well-established reproductive sequelae such as tubal infertility, ectopic pregnancy, and chronic pelvic pain, we also summarize evidence linking PID to a broad spectrum of under-recognized systemic complications, including ovarian and colorectal cancer, cardiovascular and metabolic disorders, intestinal obstruction, and mental health problems. The findings underscore the critical importance of early diagnosis, appropriate antimicrobial treatment, and comprehensive risk stratification for optimizing clinical outcomes and reducing the long-term burden of PID.

## 1 Introduction

Pelvic inflammatory disease (PID) is a serious infectious condition affecting the female upper genital tract and surrounding tissues. It primarily encompasses endometritis, salpingitis, oophoritis, pelvic peritonitis, and Tubo-ovarian abscess (TOA) (1). The principal pathogens driving PID are *Neisseria gonorrhoeae* and *Chlamydia trachomatis* (2, 3). However, the disease frequently involves complex polymicrobial infections, including various aerobic and anaerobic bacteria, *Mycoplasma genitalium*, and bacterial vaginosis-associated flora. Pathogenesis typically involves pathogens ascending through the cervical barrier, triggering an inflammatory cascade in the upper genital tract. Epidemiologically, sexually active women aged 15 to 25 years face the highest risk (2, 4). A nationally representative cross-sectional survey conducted in the U.S. from 2013–2014 revealed a self-reported PID prevalence of 4.4% among sexually experienced reproductive-aged women (3, 5), underscoring significant health disparities—particularly higher rates and complications among minority women, especially African Americans. According to global burden estimates from the Global Burden of Disease (GBD) 2021 study, approximately 500000 to 1 million new cases of PID occur annually worldwide (6). Current management adheres to guidelines from institutions like the U.S. Centers for Disease Control and Prevention (CDC) (7). This strategy relies on empirical, broad-spectrum antibiotics targeting *Neisseria gonorrhoeae*, *Chlamydia trachomatis*, and anaerobic bacteria. Recommended regimens combine ceftriaxone and doxycycline, adding metronidazole when necessary. Recent studies have suggested that integrating traditional Chinese medicine may offer enhanced symptom relief (8). Failure to promptly diagnose and treat PID may trigger devastating complications, including chronic pelvic pain, ectopic pregnancy, and tubal infertility (9).

**Figure 1:**
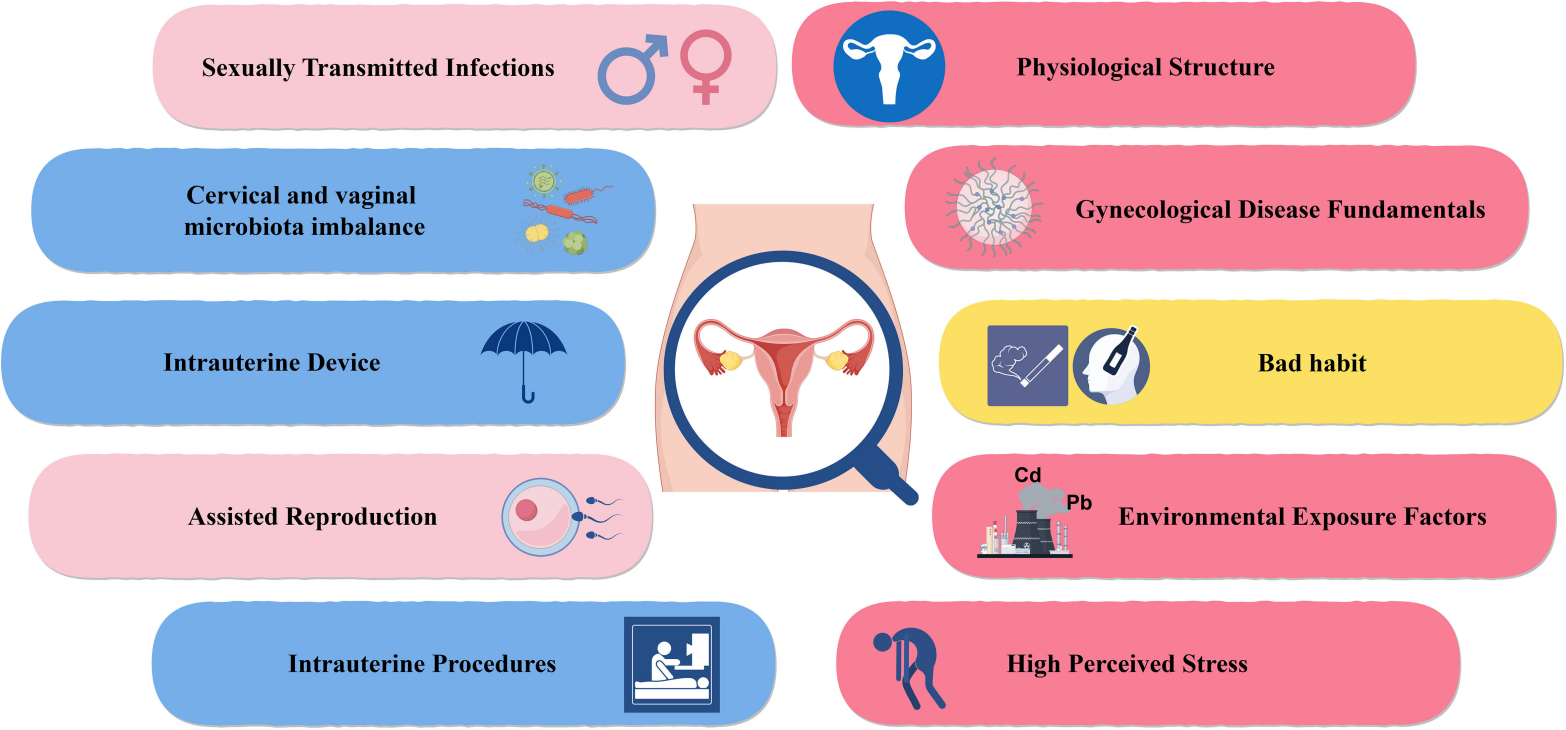
Conceptual framework of etiological factors for pelvic inflammatory disease. This figure provides a categorical overview of risk factors and does not represent a hierarchy of evidence strength.

**Figure 2:**
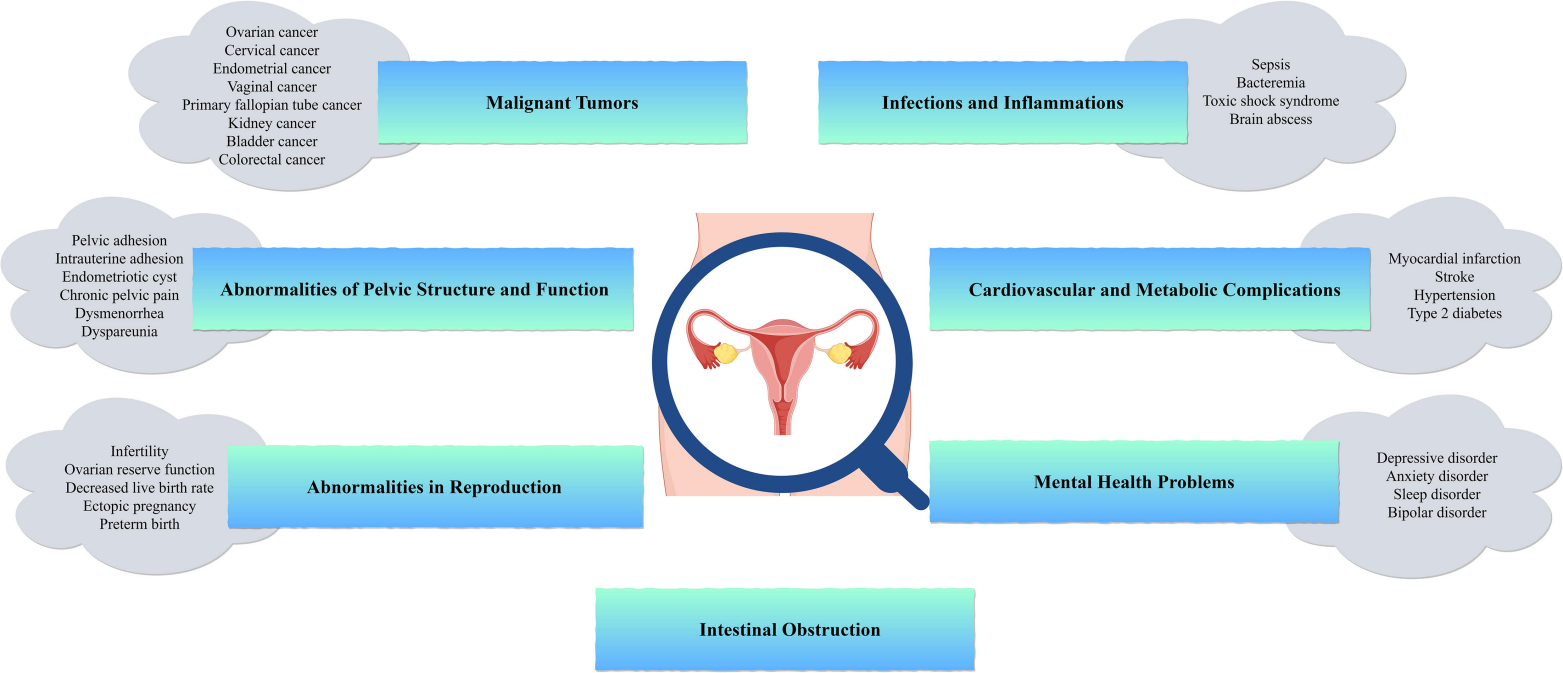
Conceptual framework of complication risks associated with pelvic inflammatory disease. This figure provides a categorical overview of potential complications and does not represent a hierarchy of evidence strength.

Ongoing research has expanded our understanding of PID etiologies and complications. Key risk factors include sexually transmitted infections, vaginal microecological disturbances, medical procedures and instrumentation, anatomical and pre-existing gynecological conditions, lifestyle choices, and environmental exposures (**Figure 1**). The complications arising from PID extend to direct reproductive system damage, pregnancy-related issues, increased risk of malignancies, severe systemic complications, and complications affecting the digestive, urinary, cardiovascular, and neurological systems, in addition to chronic health conditions (**Figure 2**). Consequently, a systematic synthesis of the etiological factors and complication risks associated with PID is of paramount importance.

## 2 Methods

This is a narrative review focusing on the inducing factors and complication risks of PID. We conducted a systematic literature search in PubMed, Embase, and Web of Science databases in December 2025, covering publications from January 2010 to December 2025, using primary search terms including “pelvic inflammatory disease”, “PID”, “risk factors”, “etiology”, “inducing factors”, “complications”, “sequelae”, “reproductive outcomes”, “infertility”, “ectopic pregnancy”, “malignancy”, “cardiovascular disease”, and “mental health” combined with Boolean operators (AND/OR). We included systematic reviews, meta-analyses, large-scale population-based cohort studies, national registry studies, and randomized controlled trials (RCTs) published in English that focused on the etiology, risk factors, or complications of PID in women of reproductive age (15–49 years), while excluding case reports (except for rare, life-threatening complications), narrative reviews without original data synthesis, non-English publications, and animal or *in vitro* experiments. Evidence was prioritized hierarchically, with systematic reviews and meta-analyses given the highest weight, followed by nationally representative cohort studies and registry data, large multicenter cohort studies, and finally single-center cohort and case-control studies; a small number of classic foundational studies published before 2010 were also included to provide essential background context. As a narrative review, this work has inherent limitations: we did not perform formal quality assessment of included studies or quantitative meta-analysis, there is a potential risk of citation bias, and the exclusion of non-English language studies may limit the generalizability of findings to non-English speaking regions.

## 3 PID inducing factors

### 3.1 Sexual transmission and vaginal microecological factors

#### 3.1.1 Sexually transmitted infections

Sexually transmitted infections (STIs) represent the primary etiological factor for PID (10, 11). Following transmission via sexual contact, these pathogens can induce ascending infections within the female upper genital tract, encompassing the uterus, fallopian tubes, ovaries, and pelvic peritoneum, particularly among sexually active women of reproductive age. Adolescents and young women are at a comparatively elevated risk due to the increased susceptibility of the columnar epithelium of their cervix to pathogen invasion (12, 13). The predominant pathogenic agents include *Chlamydia trachomatis*, *Neisseria gonorrhoeae*, *Trichomonas vaginalis*, and *Mycoplasma genitalium*, with *Chlamydia trachomatis* and *Neisseria gonorrhoeae* being identified as the principal pathogens (10, 13). A systematic review and meta-analysis of global epidemiological data estimates that over 80% of acute PID cases in high-income countries are attributable to sexually transmitted pathogens (6), corroborating that *Chlamydia trachomatis* is responsible for 23% of global STI-related PID cases and Neisseria gonorrhoeae accounts for approximately 5%, based on GBD 2021 modeling estimates (6). The incidence of PID exhibits notable age-related disparities. Women aged 20–24 constitute the highest-risk demographic, with the age-standardized prevalence rate within this group increasing most rapidly. Globally, regions characterized by a low socio-demographic index (SDI) bear the greatest disease burden of PID, whereas regions with a medium SDI experience the most pronounced rise in incidence rates (6). In 2021, there were approximately 1.009 million cases of STI-related PID among women of childbearing age worldwide, and projections indicate that this figure will continue to escalate through 2040 (6). Variations in pathogen distribution are observed across different regions. For instance, a study conducted in Israel revealed that the detection rates of *Chlamydia trachomatis* and *Neisseria gonorrhoeae* were significantly lower in the local population compared to those in European and American populations. This suggests that regional characteristics, sexual behavior patterns, and antibiotic usage practices may influence the epidemiological profile of PID (14).

#### 3.1.2 Cervicovaginal dysbiosis

Pooled estimates from observational studies suggest that cervicovaginal dysbiosis may contribute to 50–60% of idiopathic PID cases, although these estimates vary substantially by study population and diagnostic criteria (15–17). Under physiological conditions, the cervicovaginal microbiota is dominated by *Lactobacillus crispatus*, which establishes a robust biological barrier by maintaining an acidic vaginal pH, secreting antibacterial metabolites such as lactic acid and bacteriocins, and competitively adhering to epithelial cells to prevent pathogen colonization. However, cervicovaginal dysbiosis is characterized by a marked depletion of *Lactobacillus crispatus* populations and the overproliferation of anaerobic bacteria and opportunistic pathogens (17, 18), with multiple studies consistently showing that *Gardnerella*, *Fannyhessea vaginae*, *Ureaplasma urealyticum*, and *Prevotella* species are significantly enriched in PID patients, whereas the microbiota of healthy controls remains predominantly *Lactobacillus crispatus*-dominated (17). This microbial imbalance promotes PID pathogenesis through three interconnected mechanisms: it disrupts the innate biological barrier by depleting *Lactobacillus crispatus*, allowing opportunistic pathogens to penetrate the cervical mucus barrier and colonize the upper genital tract; it modulates local immune responses via metabolite signaling, suppressing protective Th1-type cellular immunity while driving chronic inflammation; and it creates a synergistic environment with sexually transmitted pathogens such as *Chlamydia trachomatis*, where high abundance of bacterial vaginosis (BV)-associated bacteria significantly increases the risk of progression from asymptomatic *Chlamydia trachomatis* infection to clinical PID. Clinically, these microbial alterations hold diagnostic potential, as a composite biomarker panel consisting of *Lactobacillus crispatus*, *Lactobacillus iners*, *Fannyhessea vaginae*, and *Ureaplasma urealyticum* has demonstrated an area under the receiver operating characteristic curve (AUC) of 0.81 for distinguishing PID cases from healthy controls (17). Furthermore, tetracycline resistance genes are highly prevalent in PID-associated microbiota, and *Lactobacillus iners* exhibits reduced susceptibility to doxycycline, a finding that may explain treatment failures in some patients (17). Taken together, cervicovaginal dysbiosis emerges as a critical modulator of PID risk and progression, acting through the interconnected pathways of barrier disruption, immune dysregulation, and synergistic interactions with sexually transmitted pathogens.

### 3.2 Factors related to medical procedures and instruments

In addition to infectious and microbiological factors, medical procedures and instrumentation represent important iatrogenic risk factors for PID.

#### 3.2.1 Intrauterine device

Intrauterine devices (IUDs) are a well-established risk factor for PID (19). The pathogenic mechanism is primarily attributed to the potential for IUD procedures to introduce pathogens from the lower genital tract into the uterine environment, thereby disrupting the cervical barrier and precipitating infections in the upper genital tract. The literature demonstrates a significant correlation between IUD usage and an elevated risk of PID, with the risk being particularly pronounced during the initial period following IUD insertion (19). Preliminary evidence from a hospital-based case-control study in Turkey revealed that the prevalence of current IUD usage among PID patients was 29.3%, markedly higher than the 12.2% observed in age-matched healthy controls (19), identifying IUDs as a major risk factor for PID. Furthermore, the risk associated with IUDs varies depending on the type of device used (20). Copper-containing IUDs may alter the vaginal microbiota, potentially elevating the risk of PID in comparison to the levonorgestrel-releasing intrauterine system (LNG-IUD), which may reduce infection risk through localized progesterone release. A study involving patients with adenomyosis reported a significantly lower incidence of PID among LNG-IUD users compared to those taking oral norethisterone (20). Furthermore, research conducted in developing countries, such as India (21), indicates that the risk of PID is exacerbated when IUD use is combined with factors like low socioeconomic status and early marriage. Conversely, a study in Jordan involving women from conservative communities did not identify a significant correlation between IUD use and PID symptom scores (21), suggesting that geographical location, population characteristics, and pathogen variability may influence the presentation of IUD-related PID.

#### 3.2.2 Assisted reproduction and intrauterine procedures

While PID associated with assisted reproductive technology (ART) is an infrequent complication, it nonetheless warrants clinical vigilance. The primary risk arises from the disruption of the cervical mucus barrier during the procedure, facilitating the ascent of opportunistic or potential pathogens from the vagina to the internal reproductive organs (22–24). The literature indicates variability in the incidence of PID across different ART procedures. Specifically, the clinical incidence of PID in intrauterine insemination (IUI) cycles is approximately 0.16–0.3 per 1000 cycles. Notably, the risk in IUI cycles utilizing the husband’s sperm (0.21 per 1000) is significantly higher than in those using donor sperm (0.03 per 1000), representing nearly a seven-fold increase in relative risk (25). It is important to highlight that the clinical manifestations of ART-related PID are frequently atypical, often presenting as mild abdominal pain or asymptomatic salpingitis, which can be easily overlooked. Nevertheless, this condition can result in fallopian tube damage, periovarian adhesions, and even ovarian abscesses, thereby adversely affecting subsequent pregnancy outcomes (26, 27). Recent studies have indicated that in patients undergoing ART who do not present high-risk factors, the routine administration of prophylactic antibiotics does not substantially decrease the incidence of PID. Prophylactic intervention is advised solely for high-risk groups, specifically those with a history of PID, bacterial vaginosis, or sexually transmitted infections (25).

Intrauterine procedures, encompassing both diagnostic and therapeutic interventions, represent significant iatrogenic risk factors for the development of PID. The underlying pathogenesis is associated with mechanical disruption of the cervical barrier, trauma to the endometrium, and the subsequent ascending colonization by vaginal flora (28, 29). Pooled data from multiple observational studies indicate that approximately 15–20% of non-sexually transmitted PID cases are directly attributable to intrauterine interventions (28, 29). Common procedures include hysterosalpingography (HSG), hysteroscopic examination and treatment, diagnostic curettage, induced abortion, and IUD insertion (28, 29). Notably, the risk of post-abortion infection can reach 1–3% (28), particularly in the absence of prophylactic antibiotic administration. A study examining patients with ovarian endometriotic cysts complicated by PID revealed that 83% of individuals in the emergency surgery cohort had previously undergone intrauterine or pelvic procedures prior to the onset of PID. The use of procedures such as endometrial cytology, *in vitro* fertilization–embryo transfer (IVF–ET), hysterosalpingography, and hysteroscopic examination was observed in 38% of the conservative treatment group (30). Furthermore, retained sutures within the uterus and conditions following vaginal hysterectomy can also contribute to the development of PID. Case studies have documented instances where a patient with a history of three cesarean sections developed PID due to retained non-absorbable sutures in the uterus (31), and another patient exhibited symptoms of PID 16 months post-vaginal hysterectomy (32). These cases suggest that intrauterine procedures may compromise the reproductive tract’s natural defense mechanisms, facilitating bacterial ascension and subsequent PID development. In recent years, there has been increasing recognition of delayed postoperative PID, which may manifest weeks to months following surgery and could be associated with occult infections or biofilm formation. Clinical studies have shown that screening and treating lower reproductive tract infections before surgery, strict aseptic techniques during surgery, and prophylactic use of antibiotics in high-risk groups can reduce the risk of intrauterine operation-related PID (28, 30).

### 3.3 Physiological structure and gynecological disease fundamentals

#### 3.3.1 Physiological structure

Physiological structural factors are critical in the development of PID. A study involving 149 women demonstrated that the longitudinal axis (48.49 ± 6.85 mm) and transverse axis (39.33 ± 5.37 mm) of the uterine body in PID patients were significantly shorter compared to those in the healthy control group, which exhibited a longitudinal axis of 53.42 ± 10.12 mm and a transverse axis of 41.95 ± 7.27 mm. Conversely, the longitudinal axis of the cervix (32.39 ± 4.47 mm) and the anterior cervical angle (128.57 ± 18.36 degrees) were significantly greater in PID patients than in the control group, which had a cervical longitudinal axis of 28.69 ± 5.21 mm and an anterior cervical angle of 109.11 ± 10.28 degrees. Binary logistic regression analysis identified the longitudinal axis of the uterine body, the longitudinal axis of the cervix, and the anterior cervical angle as significant predictors of PID. These anatomical differences may facilitate the ascent of pathogens, thereby increasing the risk of infection and the subsequent development of PID (19). In addition, post-menopausal women theoretically have a reduced susceptibility to infection due to a decrease in cervical columnar epithelial eversion, a shrinkage of the cervical transformation zone, and a thickening of cervical mucus. However, if there are conditions such as pelvic organ prolapse, the risk of developing PID will increase by 1.919 times (33).

#### 3.3.2 Gynecological disease fundamentals

The presence of underlying gynecological conditions plays a crucial role in the recurrence of PID. A study involving 98 patients who underwent laparoscopic surgery for TOA identified endometriosis as an independent risk factor for PID recurrence (34). Within the recurrence cohort, 61.9% (13 out of 21) of patients were diagnosed with endometriosis, compared to only 15.6% in the non-recurrence cohort (34). Multivariate logistic regression analysis indicated that individuals with endometriosis faced a 9.62-fold increased risk of experiencing PID recurrence relative to those without the condition (34). This heightened risk may be attributed to endometriosis-induced modifications in the pelvic environment, such as alterations in the peritoneal microenvironment that favor bacterial growth, distortion of ovarian and tubal anatomy, compromised immune responses, and elevated production of inflammatory cytokines, all of which collectively contribute to an increased likelihood of PID onset and recurrence.

### 3.4 Lifestyle habits and environmental exposure factors

#### 3.4.1 Lifestyle habits

Unhealthy lifestyle habits substantially elevate the incidence of PID through various mechanisms, including the impairment of immune function, disruption of the reproductive tract microbiome, and increased susceptibility to infections. A clear positive dose-response relationship exists between alcohol consumption and PID prevalence. Data from the U.S. National Health and Nutrition Examination Survey (NHANES) 2013–2020 indicate that, compared to abstainers, the prevalence of PID increased by 89%, 94%, and 101% among light, moderate, and heavy drinkers, respectively (35). Smoking is identified as an independent risk factor, with current and former smokers experiencing a 102% and 108% increased risk of PID, respectively (35). Additionally, vaginal douching disrupts the normal vaginal microbiota balance, with douching twice or more per month increasing the risk of endometritis by 24% (36). This disruption is associated with an elevated risk of endometrial infection by bacteria linked to bacterial vaginosis (BV). Among Australian Aboriginal women, smoking increases the risk of hospitalization for PID by 3.1 times, while low red blood cell folate levels (lowest quartile compared to highest quartile) are associated with a fourfold increase in risk (37).

#### 3.4.2 Environmental exposure factors and stress

Exposure to environmental toxins has been significantly linked to the onset of PID. Numerous studies have demonstrated a positive correlation between exposure to heavy metals, such as cadmium and lead, and the prevalence of PID. Specifically, cadmium exposure is associated with a nonlinear relationship with PID risk, whereas lead exposure demonstrates a linear relationship. No significant associations have been identified for other heavy metals, including manganese, mercury, and selenium (38). Additionally, prolonged exposure to cholinesterase inhibitor pesticides has been shown to increase the risk of PID in women by suppressing erythrocyte cholinesterase activity and inducing immunosuppression (39). Although not traditionally classified as an environmental toxin, high levels of perceived stress indirectly elevate the risk of STIs by affecting immune function and sexual behavior, thereby increasing the incidence of PID. This correlation is particularly pronounced among Black women (40).

## 4 Complications of PID

### 4.1 Malignant tumors

#### 4.1.1 Ovarian-related tumors

There exists a correlation between PID and the incidence of ovarian-related tumors (41). Research indicates that individuals with a history of PID exhibit a significantly elevated likelihood of developing ovarian cancer (42). The ovarian tumors associated with PID predominantly include borderline ovarian tumors (BOT) and epithelial ovarian cancer (43–45). A national case-control study conducted in Sweden, which included 4782 BOT patients and 45167 controls, revealed that 2.0% of BOT cases had a history of PID, compared to 1.3% in the control group. This suggests that individuals with PID have a 48% increased risk of developing BOT overall, with a 76% increased risk specifically for serous BOT. Furthermore, a dose-response relationship was identified between the number of PID episodes and the risk of serous BOT, although no significant association was found for mucinous BOT (45). A cohort study involving 441382 women in Western Australia also demonstrated that PID is associated with an increased risk of high-grade serous ovarian cancer (46). Consistent findings from multiple studies conducted in Taiwan, China, have further corroborated that PID is a contributing factor to an elevated risk of ovarian cancer (47, 48). Another study found that the risk of ovarian cancer is significantly increased in patients with PID, and this association is particularly significant in Asian populations (49, 50). Therefore, there is a significant association between PID and ovarian cancer (51–56), especially serous ovarian cancer (57, 58). However, these findings should be interpreted with caution. Potential confounding factors include smoking, parity, hormonal contraceptive use, and endometriosis, which are associated with both PID and ovarian cancer risk. Residual confounding may partially explain the observed associations, and no definitive causal relationship has been established.

#### 4.1.2 Other gynecological and urogenital system tumors

PID may increase the incidence of certain gynecological and urogenital tumors. Regarding cervical cancer, a Danish nationwide cohort study by Søgaard et al. involving 93706 PID patients showed that the Standardized Incidence Ratio (SIR) for cervical cancer was 1.0 at ≥1 year after PID diagnosis, indicating no significant association (59). However, Skapinyecz et al. observed significantly higher HPV infection rates among PID patients compared to controls, suggesting PID may increase cervical cancer risk through synergistic effects with HPV (60). For endometrial/uterine cancer, Søgaard et al. reported an SIR of 0.9 for uterine/endometrial cancer ≥1 year after PID (59). Conversely, a Taiwanese cohort study by Huang et al. showed no significant association between PID and overall uterine cancer risk. However, this association disappeared after propensity score matching, and no significant correlation was found between PID and endometrial cancer or uterine sarcoma (61). Regarding vaginal and vulvar cancers, Søgaard et al. found that ≥1 year after PID, the SIR for vaginal cancer was 2.5 and for vulvar cancer was 1.8, indicating a significantly elevated long-term risk (59). Additionally, Zardawi’s case report described three instances of primary tubal carcinoma in the context of chronic PID, suggesting chronic inflammation may promote tubal carcinogenesis through immune responses mediated by *Chlamydia trachomatis* infection (62). Regarding urinary tract tumors, Søgaard et al. reported an SIR of 1.4 for renal cancer and 1.1 for bladder cancer ≥1 year after PID, suggesting a weak association between PID and urinary tract tumors (59). Synthesizing analyses by Syed Khaja et al., PID showed significant associations with ovarian, uterine, and vaginal cancers but no significant link with cervical cancer, exhibiting overall high heterogeneity (42, 59–62). The high heterogeneity across studies may be due to differences in study design, population characteristics, and adjustment for confounding factors. Further prospective studies with rigorous confounding control are needed to validate these associations.

#### 4.1.3 Tumors of the digestive system

The relationship between PID and tumors of the digestive system is predominantly observed in colorectal cancer, with a clear distinction between short-term and long-term risk. A national cohort study conducted in Denmark revealed that within the first year following a PID diagnosis, the standardized incidence ratio (SIR) for colorectal cancer was markedly elevated at 7.6. However, beyond one year post-diagnosis, the SIR declined to 1.0, indicating no significant excess risk compared to the general population (59). Furthermore, a national cohort study in Taiwan demonstrated that the risk of colorectal cancer in PID patients increased in direct correlation with the frequency of medical consultations: the hazard ratios were 1.72 for patients with 1–12 visits and 2.84 for those with more than 12 visits. This pattern of findings strongly suggests that the short-term association between PID and colorectal cancer is most likely explained by surveillance bias rather than a causal relationship. PID diagnosis leads to increased medical attention, including more frequent physical examinations, imaging studies, and laboratory tests, which facilitates the detection of pre-existing, asymptomatic colorectal cancers that would otherwise have gone undiagnosed. The rapid normalization of risk after one year and the dose-response relationship with healthcare utilization provide strong evidence against a causal role of PID in colorectal carcinogenesis. No significant long-term causal association has been established (63).

While the association between PID and malignancy remains an area of active research, the acute infectious complications of PID are well-established and can be life-threatening.

### 4.2 Infection and inflammation

Undoubtedly, PID can trigger localized pelvic infections or abscesses (30, 34, 64–68). Furthermore, without prompt control, PID may lead to multiple severe infectious complications. Sepsis and bacteremia frequently accompany TOA, occurring in 10–15% of hospitalized PID patients. Pathogens like *Escherichia coli* and *Peptostreptococcus* can disseminate hematogenously, causing bloodstream infections definitively diagnosed via blood culture (69, 70). Though rare, toxic shock syndrome (TSS) represents a potentially fatal PID complication, triggered by pathogens such as *Group A Streptococcus* or *Clostridium perfringens*. It manifests as profound hypotension, multiple organ dysfunction (e.g., renal failure, coagulopathy), and a diffuse erythematous rash (71, 72). Brain abscess, an extremely rare PID complication (73, 74), is observed primarily in intrauterine device (IUD) users. Pathogens like *Streptococcus intermedius* or *Actinomyces* species may metastasize hematogenously to the brain, forming abscesses. Patients can present with headaches and neurological deficits, necessitating surgical drainage combined with prolonged antibiotic therapy (e.g., ceftriaxone). Only a handful of global cases exist in the literature. These grave complications underscore the critical need for early PID intervention, particularly among TOA patients, IUD users, and immunocompromised individuals, who demand heightened vigilance against the risk of infection spread.

### 4.3 Abnormalities in the structure and function of the pelvic cavity

PID can induce multifaceted abnormalities in pelvic structure and function, primarily through chronic inflammation and cervicovaginal dysbiosis caused by ascending pathogen infection. Structurally, PID induces selective loss of ciliated epithelial cells in the fallopian tubes, obstructing egg transport and increasing the risk of tubal infertility or ectopic pregnancy (75). Concurrently, the inflammatory process promotes pelvic adhesions and endometriotic cyst formation, further distorting pelvic anatomy (76). Functionally, PID-associated reproductive tract dysbiosis disrupts the immune equilibrium of the endometrial microbiota. Concurrently, excessive production of pro-inflammatory cytokines such as IL-1β, IL-6, and TNF-α enhances the peritoneal adhesion capacity of ectopic endometrial tissue, ultimately manifesting as chronic pelvic pain, dysmenorrhea, and dyspareunia (75, 76). Furthermore, PID exhibits a significant association with endometriosis risk, with patients experiencing PID showing a higher likelihood of developing endometriosis (75). Endometriotic cysts further increase the risk of antibiotic treatment failure and surgical intervention in PID patients, creating a vicious cycle (77). Prolonged chronic inflammation may also cause uterine contractility dysfunction and intrauterine adhesions, further impairing reproductive function (76, 78).

### 4.4 Abnormalities in reproduction

PID is a major cause of female infertility and reduced fertility. Cui et al. found that serum anti-Müllerian hormone levels were decreased in patients with bilateral tubal obstruction, suggesting chronic PID may impair ovarian reserve function (79). A systematic review of cohort studies found that approximately 18% of women develop tubal infertility following a single episode of PID (80). The risk of infertility increases with the severity of tubal damage, reaching up to 30% in cases of severe tubal injury. Recurrent infections significantly elevate the risk of infertility (80). Preliminary evidence from a single-center retrospective cohort study in Thailand revealed that 25.5% of hospitalized PID patients developed infertility during a median follow-up of 69 months, with only 31.9% achieving live births (81). An analysis by Anyalechi et al. based on U.S. NHANES data revealed a 24.2% infertility prevalence among women reporting PID treatment, significantly higher than those without PID treatment. This association was most pronounced among young women aged 18–29 years (82). This evidence indicates that PID significantly reduces female fertility through mechanisms such as tubal damage and diminished ovarian reserve. Disease severity, recurrence, and age are key factors influencing prognosis.

Additionally, PID is a risk factor for ectopic pregnancy and preterm birth. A retrospective cohort study by Huang et al. based on Taiwan’s National Health Insurance database (83) showed that during a 10-year follow-up period involving 30450 PID patients and 91350 controls, the cumulative incidence of preterm birth among PID patients was 1.84%, significantly higher than the 1.63% observed in those without PID. The cumulative incidence of ectopic pregnancy among PID patients was 0.05%, also higher than the 0.04% observed in those without PID. After adjusting for confounding factors via multivariate Cox proportional hazards regression analysis, the risk of preterm birth among PID patients was 1.864 times higher than in those without PID, and the risk of ectopic pregnancy was 2.121 times higher (83). This study further confirms that a history of PID is an independent risk factor for preterm birth and ectopic pregnancy, with particularly elevated risks among adolescent females aged 12–19 years. This large-scale population study provides crucial evidence linking PID to adverse pregnancy outcomes in Asian populations, suggesting clinicians should conduct careful assessment and monitoring during pregnancy in PID patients.

### 4.5 Intestinal obstruction

Intestinal obstruction caused by PID is a rare but significant complication (84), primarily resulting from inflammatory adhesion formation, mechanical compression of the transverse omentum, and perihepatic adhesions associated with Fitz-Hugh-Curtis syndrome. Sia et al. reported a 19-year-old patient with PID caused by *Chlamydia trachomatis* infection. Intraoperative findings revealed band-like adhesions between the greater omentum and small bowel mesentery, along with “string-like” perihhepatic adhesions, leading to a diagnosis of small bowel obstruction (85). Ahmed et al. reported a 38-year-old patient with TOA complicating IUD-associated PID. CT revealed multiple pelvic abscesses compressing the sigmoid colon, causing obstruction that resolved after conservative management (antibiotics, gastrointestinal decompression) (86). Haumann et al. described a 27-year-old patient who developed obstruction early after acute *Chlamydia trachomatis* infection due to a 360° adhesive band encircling the jejunum, emphasizing the importance of early laparoscopic exploration (87). Al-Ghassab et al. reported a 32-year-old patient with no clear history of PID, but laparoscopy revealed small bowel adhesions, “string-like” perihilar adhesions, and hydrosalpinx of the left fallopian tube, suggesting subclinical PID can cause delayed obstruction (88). A literature review by Chan et al. summarized 14 cases of PID-related obstructive complications, including 6 cases of intestinal obstruction. Mechanisms involved direct compression by the transverse omentum, formation of inflammatory adhesions, and internal hernias due to perihepatic adhesions in Fitz-Hugh-Curtis syndrome (65). Khan et al. reported a unique case in a patient over 40 years old who developed adhesive small bowel obstruction requiring exploratory laparotomy after multiple hospitalizations for PID (89).

### 4.6 Cardiovascular and metabolic abnormalities

Several large-scale population studies have reported associations between PID and cardiovascular/metabolic complications, although these findings remain inconsistent across populations. The observed associations may be explained by multiple factors: (1) shared risk factors such as smoking, obesity, socioeconomic status, and sexual behavior; (2) detection bias due to increased healthcare utilization among PID patients; and (3) residual confounding from unmeasured variables. While chronic inflammation is a biologically plausible mechanism, no causal relationship has been definitively established. A cohort study conducted by Liou et al., utilizing data from China Taiwan’s National Health Insurance database, demonstrated that among 68453 PID patients monitored over a three-year period, the risk of myocardial infarction (MI) was significantly elevated compared to the control group. This risk was particularly pronounced in individuals over 55 years of age. Further analysis revealed that the risk of MI was especially heightened in patients with acute PID (90). Subsequent research by Hsu et al. categorized genital tract infections and found that upper genital tract infections were associated with an adjusted Hazard Ratio (HR) for MI of 2.88, which was significantly higher than that for lower genital tract infections. This finding suggests a positive correlation between the severity of infection and cardiovascular risk (91). In a study by Chen et al. focusing on stroke, involving 64515 PID patients, there was a 1.63-fold increase in stroke risk after three years of follow-up compared to controls. The risk of ischemic stroke was notably increased, whereas no significant difference was observed for hemorrhagic stroke (92). However, a retrospective matched cohort study by Okoth et al. using the U.K. THIN database found no significant association between PID history and composite cardiovascular disease or its subtypes, though it confirmed independent associations between PID and risks of hypertension and type 2 diabetes (93).

### 4.7 Mental health problems

PID, a prevalent infectious condition among females, not only induces considerable physical discomfort but also significantly affects patients’ mental well-being. A comprehensive nationwide retrospective cohort study utilizing data from Taiwan’s National Health Insurance Research Database revealed that individuals with PID have a markedly elevated risk of developing subsequent mental disorders compared to a control group (94). Specifically, the adjusted risk ratios for the onset of bipolar disorder, depressive disorder, anxiety disorder, and sleep disorder in PID patients were 2.671, 2.173, 2.006, and 2.251, respectively, all of which were significantly higher than those observed in the control group without PID. Throughout the study period, 9.0% of individuals with PID and 4.7% of controls were diagnosed with mental disorders. Among those with PID, the most prevalent subsequent mental disorders were depressive disorder (6.0%), anxiety disorder (4.3%), and sleep disorder (3.3%), with bipolar disorder affecting 0.6% (94). The findings of this study suggest that PID may elevate the risk of mental disorders by initiating inflammatory responses, causing tissue damage and adhesions, and consequently leading to physical discomfort such as chronic pelvic pain, which diminishes quality of life (94). Furthermore, PID and mental disorders may share common pathophysiological mechanisms involving genetic, environmental, hormonal, or neuropsychological factors (94). Importantly, reverse causality cannot be ruled out: pre-existing mental health conditions may increase the risk of STI acquisition and PID through impaired immune function and risky sexual behavior. Prospective studies with baseline mental health assessments are needed to clarify the temporal relationship.

## 5 Discussion

This narrative review systematically synthesizes both well-established consensus evidence and emerging preliminary evidence on the inducing factors and complication risks of pelvic inflammatory disease (PID). Well-established inducing factors include sexually transmitted infections, cervicovaginal dysbiosis characterized by depletion of Lactobacillus crispatus and overproliferation of anaerobic bacteria, intrauterine device insertion, other intrauterine procedures, underlying endometriosis, and adverse lifestyle habits such as smoking, excessive alcohol consumption, and frequent vaginal douching. Well-established complications include tubal infertility, ectopic pregnancy chronic pelvic pain, tubo-ovarian abscess, sepsis and bacteremia, toxic shock syndrome, pelvic adhesions, and preterm birth. In contrast, emerging or less definitively established associations in inducing factors include specific anatomical variations, pelvic organ prolapse in postmenopausal women, environmental exposures to heavy metals and cholinesterase inhibitor pesticides, high perceived stress, and the differential risk between copper-containing intrauterine devices and levonorgestrel-releasing intrauterine systems. For complications, emerging evidence suggests potential links to ovarian cancer, vaginal and vulvar cancers, primary fallopian tube carcinoma, intestinal obstruction secondary to inflammatory adhesions and Fitz-Hugh-Curtis syndrome, cardiovascular events, metabolic disorders, diminished ovarian reserve, and a broad spectrum of mental health disorders including depression, anxiety, sleep disturbances, and bipolar disorder. Notably, the observed short-term increase in colorectal cancer risk after PID diagnosis is most likely attributable to surveillance bias rather than a causal relationship. These emerging associations require rigorous validation in large-scale, long-term prospective cohort studies with comprehensive confounding control before they can be integrated into routine clinical risk stratification and management guidelines.

Despite current progress, the clinical management of PID remains challenged by delayed diagnosis due to nonspecific symptoms and the rising prevalence of antibiotic resistance. Improving early recognition, advancing rapid pathogen detection, and implementing individualized antimicrobial strategies are therefore critical to enhance patient outcomes. A key limitation of existing evidence is the difficulty in fully controlling for confounding and bias, particularly in systemic outcomes. Furthermore, while this review includes both well-established and emerging associations, the strength of evidence varies considerably across different outcomes. Clinical recommendations should be based primarily on well-established findings, while emerging associations should be interpreted with caution and require further validation in future research.

Future research should directly address the key gaps identified in this review by conducting prospective cohort studies to validate the causal role of cervicovaginal dysbiosis in PID pathogenesis and evaluate probiotic interventions for recurrence prevention, addressing the current lack of longitudinal data on microbiome-PID interactions; performing large-scale, long-term prospective studies with rigorous control for confounding factors (smoking, socioeconomic status, healthcare utilization) to clarify causal relationships between PID and systemic outcomes including ovarian cancer and cardiovascular disease, which currently rely predominantly on retrospective data; developing and validating machine learning-based risk prediction models integrating clinical, microbiological, and demographic data to identify patients at high risk of severe complications, addressing the absence of standardized risk stratification tools in clinical practice; strengthening epidemiological surveillance in low- and middle-income countries, where the global burden of PID is highest but population-level data remain most limited; and conducting randomized controlled trials to evaluate the efficacy and safety of optimized antimicrobial regimens and adjunctive therapies for PID management, addressing the rising challenge of antibiotic resistance.
